# Technostress and Insulin Resistance Risk: A Cross-Sectional Analysis of 104,175 Spanish Workers Using TyG, TyG-BMI, METS-IR, and SPISE Indices

**DOI:** 10.3390/medsci14050545

**Published:** 2026-09-03

**Authors:** Marta González Rivas, Ángel Arturo López-González, Diego González Carrasco, Carla Busquets-Cortés, María Teófila Vicente-Herrero, José Ignacio Ramírez-Manent

**Affiliations:** 1 ADEMA University School, University of the Balearic Islands, 07009 Palma, Spain; 2 Primary Care, Balearic Health Service, 07010 Palma, Spain

**Keywords:** technostress, insulin resistance, triglyceride–glucose (TyG) index, triglyceride–glucose body mass index (TyG-BMI), metabolic score for insulin resistance (METS-IR), single-point insulin sensitivity estimator (SPISE), occupational health

## Abstract

**Background:** Technostress has emerged as a growing occupational health concern in increasingly digitalized workplaces. Although its psychological consequences have been extensively investigated, little is known about its potential association with metabolic health and insulin resistance. This study aimed to evaluate the relationship between technostress and insulin resistance risk using four validated surrogate markers in a large cohort of Spanish workers. **Methods:** A cross-sectional study was conducted among 104,175 Spanish workers who underwent routine occupational health assessments. Technostress was assessed using a 15-item questionnaire covering five technostress dimensions and was analyzed using four operational categories (low, moderate, high, and very high). Insulin resistance risk was assessed using the triglyceride–glucose (TyG) index, TyG-body mass index (TyG-BMI), the metabolic score for insulin resistance (METS-IR), and the single-point insulin sensitivity estimator (SPISE). Sociodemographic characteristics, lifestyle habits, anthropometric measurements, and biochemical parameters were also recorded. Modified Poisson regression with robust variance estimation was used to estimate crude and adjusted prevalence ratios (PRs) for increased insulin resistance risk according to technostress level. Firth penalized logistic regression was additionally performed as a sensitivity analysis to address sparse-data bias and separation. **Results:** Higher technostress levels were associated with progressively less favorable anthropometric and metabolic profiles. The prevalence of increased insulin resistance risk rose significantly across technostress categories for all markers evaluated (*p* < 0.001). For TyG, prevalence increased from 3.8% in the low technostress group to 54.3% in the very high technostress group. Corresponding increases were observed for TyG-BMI (0.2% to 78.2%), METS-IR (0.0% to 45.2%), and elevated SPISE-IR values (0.0% to 57.5%). After adjustment for age, sex, educational level, physical activity, adherence to the Mediterranean diet, and smoking status, higher technostress remained associated with a higher prevalence of increased insulin resistance risk. A clear graded cross-sectional pattern was observed, with the strongest associations found among workers reporting very high technostress. **Conclusions:** Higher technostress was associated with a higher prevalence of increased insulin resistance risk according to TyG, the primary outcome, with a clear graded cross-sectional pattern across technostress categories. Associations were also observed for the secondary BMI-containing indices (TyG-BMI, METS-IR, and SPISE-IR), which should be interpreted as complementary rather than independent evidence. Given the cross-sectional design and the pronounced clustering of technostress with adiposity and lifestyle characteristics, residual confounding cannot be excluded and causal inference is not possible.

## 1. Introduction

The rapid digitalization of contemporary workplaces has transformed work organization, communication, and task performance. Although information and communication technologies (ICTs) offer substantial benefits in productivity and flexibility, their widespread integration into daily work has also introduced new psychosocial demands that may adversely affect employee well-being and occupational health [1,2,3,4].

Among these emerging challenges, technostress has become an increasingly relevant occupational health concern. Originally described by Brod as a modern disease of adaptation resulting from an inability to cope effectively with computer technologies, technostress is currently defined as a specific form of stress arising when technological demands exceed an individual’s resources, competencies, or coping capacities [5,6,7]. Contemporary models conceptualize technostress as a multidimensional construct comprising techno-overload, techno-invasion, techno-complexity, techno-insecurity, and techno-uncertainty. These dimensions reflect different ways in which digital technologies may generate psychological strain, including excessive workload, constant connectivity, difficulties adapting to technological change, concerns regarding technological obsolescence, and the continuous need to acquire new digital skills [6,7,8].

The widespread adoption of remote and hybrid working arrangements has increased workers’ dependence on digital technologies and blurred the boundaries between professional and personal life [9,10,11]. Greater exposure to information overload, frequent interruptions, accelerated work rhythms, and expectations of constant availability has consequently raised concerns regarding the potential health implications of technology-related stress [10,11].

Recent evidence from highly digitalized occupational settings further indicates that technostress remains a relevant workplace health concern, with technological usability, familiarity with digital tools, and the conditions under which these technologies are implemented influencing workers’ perceived technostress [12].

Recent evidence from Spanish teleworkers also indicates that technostress-related perceptions have continued to evolve alongside changes in ICT use, reinforcing the relevance of monitoring technology-related psychosocial risks in increasingly digitalized work environments [13].

Most previous research on technostress has focused on psychological and occupational outcomes, including burnout, anxiety, depressive symptoms, reduced job satisfaction, sleep disturbances, impaired recovery, and poorer work–life balance [14,15,16]. However, its potential relationship with metabolic health and insulin resistance remains comparatively underexplored. Recent systematic evidence further indicates that physiological research on technostress remains limited, with relatively few studies incorporating objective biomarkers such as cortisol, salivary α-amylase, cardiovascular measures, or inflammatory markers, underscoring the need to extend technostress research beyond self-reported psychological outcomes [6].

Accumulating evidence indicates that chronic psychosocial stress contributes substantially to the development of cardiometabolic disorders. Persistent activation of stress-response systems promotes neuroendocrine, inflammatory, and behavioral changes that may adversely affect metabolic health [17,18,19]. In particular, chronic activation of the hypothalamic–pituitary–adrenal (HPA) axis and the sympathetic nervous system increases the secretion of cortisol and catecholamines. Although these responses are adaptive during acute stress, prolonged activation may promote visceral adiposity, dyslipidemia, endothelial dysfunction, impaired glucose metabolism, and increased cardiometabolic risk [18,19,20].

Among the various metabolic abnormalities associated with chronic stress, insulin resistance (IR) occupies a central position. IR is characterized by a diminished biological response of peripheral tissues to insulin and is widely recognized as a fundamental pathophysiological mechanism underlying type 2 diabetes mellitus (T2DM), metabolic syndrome, metabolic dysfunction-associated steatotic liver disease (MASLD), and cardiovascular disease [21,22,23]. Importantly, IR often develops years before the onset of overt clinical disease and is therefore considered one of the earliest detectable manifestations of cardiometabolic dysfunction [24]. In addition to impairing glucose homeostasis, IR contributes to chronic low-grade inflammation, atherogenic dyslipidemia, hypertension, endothelial dysfunction, and accelerated atherosclerosis, thereby increasing long-term cardiovascular risk [22,23,24].

The relationship between chronic stress and insulin resistance is biologically plausible and supported by growing evidence. Elevated cortisol concentrations promote hepatic glucose production and impair insulin-mediated glucose uptake in peripheral tissues, while inflammatory cytokines interfere with intracellular insulin signaling pathways [18,19,25]. Moreover, individuals exposed to prolonged stress frequently adopt unhealthy lifestyle behaviors, including physical inactivity, poor dietary habits, inadequate sleep, smoking, and increased sedentary behavior, all of which contribute to reduced insulin sensitivity [20,26]. Because technostress shares many characteristics with other forms of chronic occupational stress, similar physiological and behavioral mechanisms may explain its potential association with insulin resistance.

Although chronic psychosocial stress has been associated with adverse cardiometabolic outcomes, the magnitude of these associations reported in epidemiological studies has generally been modest. A systematic review and meta-analysis by Kuo et al. found a modest overall association between psychological stress and metabolic syndrome, with similarly moderate associations observed for occupational stress [27]. Evidence specifically addressing the physiological consequences of technostress remains even more limited. In the first prospective study examining the biological effects of workplace technostress, Kaltenegger et al. found no significant prospective association between technostress and C-reactive protein and, unexpectedly, observed an inverse association with hair cortisol concentration [28]. These findings suggest that the physiological correlates of technostress may be more complex than predicted by a simple chronic-stress model and highlight the limited and heterogeneous nature of the available biological evidence. Importantly, however, prospective evidence specifically examining technostress in relation to insulin resistance remains scarce, providing a rationale for investigating this association using metabolic indicators of insulin sensitivity.

Accurate assessment of insulin resistance is essential when investigating these relationships. Although the hyperinsulinemic–euglycemic clamp remains the gold-standard method for measuring insulin sensitivity, its complexity, invasiveness, and cost limit its applicability in large epidemiological studies [29]. Likewise, the Homeostasis Model Assessment of Insulin Resistance (HOMA-IR) requires insulin measurements that are not routinely available in many occupational health settings [30]. Consequently, several surrogate markers based on routinely collected anthropometric and biochemical variables have been developed and validated.

Among these, the triglyceride–glucose (TyG) index has emerged as one of the most extensively studied markers of insulin resistance and has demonstrated strong associations with T2DM, metabolic syndrome, MASLD, and cardiovascular disease [31]. The triglyceride–glucose–body mass index (TyG-BMI) incorporates information regarding adiposity and may further improve metabolic risk stratification [32]. More recently, the Metabolic Score for Insulin Resistance (METS-IR) and the Single-Point Insulin Sensitivity Estimator (SPISE) have shown promising performance as non-insulin-based indicators of insulin resistance and have demonstrated associations with multiple cardiometabolic outcomes in diverse populations [33,34,35].

Recent evidence from large occupational cohorts has further supported the applicability of these non-insulin-based surrogate indices in working populations, with TyG, TyG-BMI, METS-IR, and SPISE showing consistent associations with occupational and lifestyle-related metabolic risk factors [34].

Despite the biological plausibility linking technostress and insulin resistance, evidence examining this relationship remains scarce. Most available studies have focused primarily on psychological outcomes, whereas the potential metabolic consequences of technology-related stress have received comparatively little attention. Although these surrogate indices have previously been applied in large occupational cohorts to examine metabolic risk in relation to occupational and lifestyle factors, their association with technostress remains insufficiently characterized. The specific contribution of the present study is therefore the evaluation of technostress as the exposure of interest in relation to these established metabolic indices within a large occupational population.

Understanding whether technostress contributes to impaired insulin sensitivity may provide important insights into the broader health consequences of workplace digitalization. Such knowledge could help identify workers at increased cardiometabolic risk and inform preventive interventions aimed at reducing both psychosocial and metabolic burdens in increasingly technology-dependent occupational environments.

Therefore, the aim of the present study was to investigate the association between technostress and insulin resistance risk assessed using four validated surrogate markers—TyG, TyG-BMI, METS-IR, and SPISE—in a large cohort of Spanish workers. We hypothesized that higher levels of technostress would be associated with a higher prevalence of increased insulin resistance risk after adjustment for measured sociodemographic characteristics and lifestyle factors.

## 2. Materials and Methods

### 2.1. Study Design and Population

A cross-sectional study was conducted using data obtained from routine occupational health examinations performed between January 2021 and December 2024 in several regions of Spain. These examinations were carried out by trained occupational health professionals as part of periodic workplace health surveillance programs established under Spanish occupational health regulations. The study was designed and reported in accordance with the Strengthening the Reporting of Observational Studies in Epidemiology (STROBE) recommendations for observational studies [36].

The study population consisted of actively employed workers who underwent a routine occupational health assessment during the study period. Data were collected using standardized questionnaires, anthropometric measurements, and fasting biochemical determinations performed according to homogeneous occupational health protocols.

**Figure 1:**
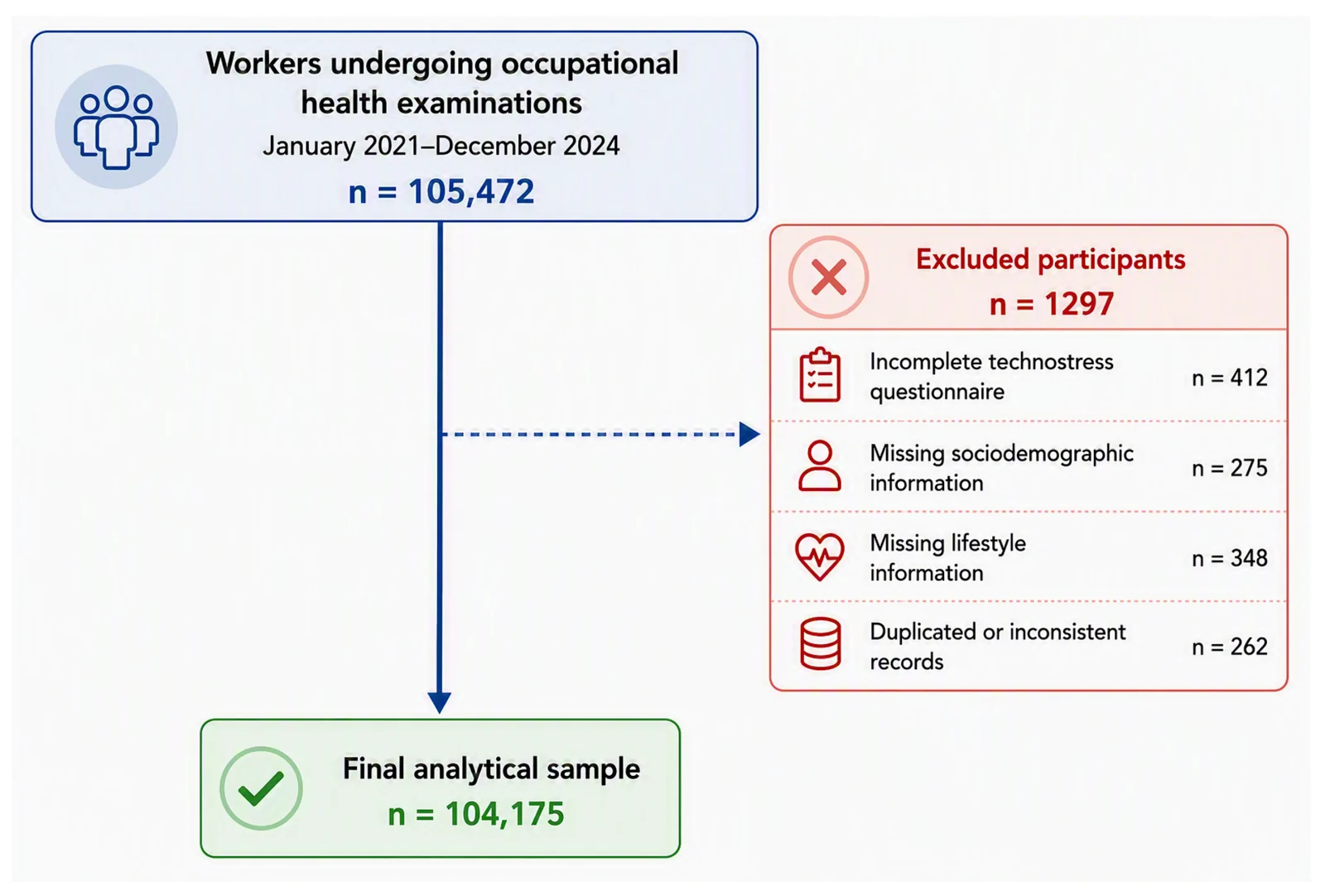
Flowchart of participant selection.

A total of 105,472 workers underwent occupational health examinations during the study period and were initially considered for inclusion. Of these, 1297 workers (1.23%) were excluded: 412 because of an incomplete technostress questionnaire, 275 because of missing sociodemographic information, 348 because of missing lifestyle information, and 262 because of duplicated or inconsistent records. After these exclusions, the final complete-case analytical sample comprised 104,175 workers. The participant selection process is summarized in Figure 1.

### 2.2. Sociodemographic Variables

Information on sex, age, educational attainment, and occupational social class was collected using standardized questionnaires administered during the occupational health examination.

Sex was classified as male or female. Age was analyzed as a continuous variable in the multivariable models to provide more flexible control for age-related confounding. For descriptive purposes, age was additionally grouped into five categories: 20–29, 30–39, 40–49, 50–59, and 60–69 years.

Educational attainment was categorized into three groups: primary education, secondary education, and university education.

Occupational social class was assigned according to the participant’s occupational category, following the classification proposed by the Spanish Society of Epidemiology based on the Spanish National Classification of Occupations 2011 (CNO-11). This classification groups occupations according to qualification level, responsibility, and employment status, and has been widely used in Spanish epidemiological studies of social inequalities in health [37].

### 2.3. Lifestyle Variables

Physical activity was assessed using the short version of the International Physical Activity Questionnaire (IPAQ-SF), a widely used instrument for estimating habitual physical activity in population-based studies [38,39]. Participants were classified as physically active or physically inactive according to their regular engagement in physical activity, based on weekly energy expenditure expressed in metabolic equivalent task minutes per week (MET-min/week). In the analytical dataset available for the present study, physical activity was retained as a dichotomous variable (active versus inactive), whereas the original continuous MET-min/week values were not available for reanalysis. Consequently, physical activity was included in the multivariable models as a dichotomous covariate.

Adherence to the Mediterranean diet was evaluated using the 14-item Mediterranean Diet Adherence Screener (MEDAS), originally developed within the PREDIMED framework and validated for the rapid assessment of adherence to the Mediterranean dietary pattern [40,41]. The questionnaire evaluates the consumption frequency of key components of the Mediterranean diet, including olive oil, vegetables, fruits, legumes, fish, nuts, and other characteristic food groups. Each item contributes one point when the established criterion is fulfilled, resulting in a total score ranging from 0 to 14 points. In the analytical dataset available for the present study, Mediterranean diet adherence was retained as a dichotomous variable (high versus low-to-moderate adherence); the original continuous MEDAS score was not available for reanalysis. Consequently, Mediterranean diet adherence was included in the multivariable models as a dichotomous covariate.

Smoking status was obtained by self-report. Participants who reported current tobacco consumption at the time of the examination were classified as smokers, whereas all remaining participants were classified as non-smokers.

### 2.4. Assessment of Technostress

Technostress was assessed using a 15-item short-form questionnaire covering the five dimensions commonly described within the technostress-creators framework: techno-overload, techno-invasion, techno-complexity, techno-uncertainty, and techno-insecurity. Each dimension was represented by three items addressing technology-related demands in the occupational environment. The complete wording of the 15 items and their corresponding dimensions is provided in Supplementary Table S1.

Participants indicated their level of agreement with each statement using a five-point Likert scale ranging from 1 (strongly disagree) to 5 (strongly agree). The occupational assessment system calculated an overall technostress score as the arithmetic mean of the 15 items, theoretically ranging from 1 to 5, with higher scores representing greater perceived technology-related stress. This score was subsequently classified into four operational categories for storage in the analytical dataset.

For categorical analyses and descriptive presentation, the overall mean score was divided into four analytical ranges: low (1.0–2.0), moderate (2.1–3.0), high (3.1–4.0), and very high (4.1–5.0) technostress. These categories represent pragmatic subdivisions of the 1–5 response range used to facilitate descriptive comparison across increasing levels of perceived technostress; they were not derived from externally validated clinical, diagnostic, or prognostic thresholds. Accordingly, the categorical analyses should be interpreted as comparisons across operational exposure groups rather than validated technostress severity categories.

The specific 15-item short form used in this study was developed for routine occupational health assessment based on the five dimensions of the technostress-creators framework, but it has not undergone formal external psychometric validation against an established technostress instrument. The analytical dataset retained only the final technostress category and not the underlying individual continuous score or item-level responses; consequently, analyses treating technostress as a continuous exposure, internal consistency coefficients, item-level analyses, and confirmatory factor analyses could not be performed retrospectively in the present sample. Test–retest reliability and concurrent or criterion validity against an external technostress measure were also unavailable. Accordingly, the technostress measure should be regarded as an operational occupational assessment tool rather than a fully validated psychometric instrument, and this limitation was considered when interpreting the findings.

For the categorical analyses, low technostress was used as the reference category.

### 2.5. Anthropometric and Biochemical Measurements

Anthropometric measurements were performed by trained health personnel during the occupational health examination. Body weight was measured in light clothing and without shoes using calibrated scales. Height was measured using a stadiometer, with participants standing upright and barefoot. Body mass index (BMI) was calculated as weight in kilograms divided by height in meters squared.

Blood samples were obtained after overnight fasting as part of the routine occupational health examination. Fasting glucose, total cholesterol, HDL cholesterol, LDL cholesterol, and triglycerides were determined using standardized enzymatic laboratory methods. All biochemical parameters were expressed in mg/dL.

### 2.6. Insulin Resistance Indicators

Insulin resistance risk was assessed using four non-insulin-based surrogate markers: the triglyceride–glucose index (TyG), triglyceride–glucose–body mass index (TyG-BMI), Metabolic Score for Insulin Resistance (METS-IR), and Single-Point Insulin Sensitivity Estimator (SPISE). These indices were selected because they can be calculated from routinely available anthropometric and biochemical variables and are suitable for large epidemiological and occupational health studies [42,43,44,45].

The TyG index was calculated as ln [fasting triglycerides (mg/dL) × fasting glucose (mg/dL)/2]. TyG-BMI was calculated as TyG × BMI (kg/m^2^). METS-IR was calculated as ln [(2 × fasting glucose [mg/dL]) + fasting triglycerides (mg/dL)] × BMI/ln [HDL cholesterol (mg/dL)]. SPISE was calculated as 600 × HDL cholesterol^0.185^/[fasting triglycerides^0.2^ × BMI^1.338^], with higher SPISE values indicating greater insulin sensitivity and lower values indicating lower insulin sensitivity.

For the main analyses, each insulin resistance marker was evaluated both as a continuous and as a categorical variable. Increased insulin resistance risk was defined according to the operational thresholds applied in the analytical dataset: TyG ≥ 8.70 in women and ≥8.80 in men, TyG-BMI ≥ 255, METS-IR ≥ 50, and SPISE-IR ≥ 2.21. For categorical insulin resistance risk analyses, SPISE was transformed into the resistance-oriented index SPISE-IR according to the formula SPISE-IR = 10/SPISE. Thus, higher SPISE values indicate greater insulin sensitivity, whereas higher SPISE-IR values indicate greater insulin resistance. Elevated insulin resistance risk was defined as SPISE-IR ≥ 2.21. The thresholds used for categorical risk classification were selected from previously published epidemiological and validation studies but should be regarded as operational rather than universal diagnostic cut-offs. The sex-specific TyG thresholds (≥8.70 in women and ≥8.80 in men) are supported by previous studies in adult populations in which values around 8.7–8.8 showed good discrimination of adverse metabolic phenotypes. The METS-IR threshold of ≥50 was based on the original validation study, in which METS-IR showed good agreement with insulin sensitivity assessed using the euglycemic–hyperinsulinemic clamp and values in the highest quartile (>50.39) identified individuals at substantially increased risk of incident type 2 diabetes. The thresholds applied to TyG-BMI and SPISE-IR were derived from previously proposed epidemiological risk classifications; however, unlike METS-IR and the original SPISE construct, these specific dichotomization thresholds have not been validated against the euglycemic–hyperinsulinemic clamp in a population directly comparable to Spanish working adults. Therefore, the categorical thresholds were used for epidemiological risk stratification rather than clinical diagnosis. Because optimal cut-offs for these surrogate indices vary according to population, age, sex, reference standard, and clinical outcome, continuous values of all four indices were also analyzed and were given greater interpretative emphasis to reduce dependence on specific thresholds [42,43,44,45]. Importantly, the specific categorical thresholds applied in the present study have not been jointly validated against a direct reference measure of insulin resistance, such as the euglycemic–hyperinsulinemic clamp, in a population specifically representative of Spanish working adults. Accordingly, categorical analyses should be interpreted as epidemiological risk stratification rather than as clinical diagnosis of insulin resistance.

### 2.7. Outcome Variables

The primary outcome was increased insulin resistance risk according to the TyG index, which does not incorporate BMI in its calculation. TyG-BMI, METS-IR, and SPISE-IR were considered secondary outcomes because these indices incorporate BMI and therefore partly reflect adiposity-related metabolic risk. Accordingly, these secondary indices were used as complementary measures and were not interpreted as providing independent evidence of metabolic dysfunction beyond adiposity. Given the sparse number of high-risk events in the low-technostress reference category for some dichotomized secondary outcomes, continuous values of TyG, TyG-BMI, METS-IR, and SPISE were additionally analyzed and given particular emphasis because they avoid dependence on specific risk thresholds and reduce the statistical instability associated with sparse categorical outcomes. Correlations among the four continuous surrogate indices were assessed using Spearman correlation coefficients and are reported in Supplementary Table S4.

### 2.8. Statistical Analysis

Continuous variables were expressed as means and standard deviations (SDs), whereas categorical variables were presented as absolute frequencies and percentages.

First, sociodemographic and lifestyle characteristics were described according to technostress categories. Overall differences across the four technostress categories were assessed using one-way analysis of variance (ANOVA) for continuous variables and Pearson’s chi-square test for categorical variables. The reported *p*-values correspond to these overall between-group tests.

Given the pronounced differences in participant characteristics across technostress categories, standardized mean differences (SMDs) were additionally calculated to quantify covariate imbalance. For interpretability, SMDs comparing the low and very-high technostress groups were calculated for the principal sociodemographic and lifestyle covariates. Absolute SMD values ≥ 0.10 were considered indicative of meaningful imbalance.

Second, mean values of TyG, TyG-BMI, METS-IR, and SPISE were compared across the four technostress categories using one-way ANOVA. The corresponding *p*-values represent overall tests of differences in mean values across the four technostress categories.

Third, the prevalence of increased insulin resistance risk according to TyG, TyG-BMI, METS-IR, and SPISE-IR was calculated for each technostress category. Overall differences in prevalence across the four technostress categories were assessed using Pearson’s chi-square test. The corresponding *p*-values represent overall chi-square tests rather than pairwise comparisons between individual technostress categories.

Fourth, modified Poisson regression models with robust variance estimation were constructed to evaluate the association between technostress and increased insulin resistance risk. Crude prevalence ratios (PRs) and 95% confidence intervals (95% CIs) were initially estimated, followed by multivariable models adjusted for potential confounders. Prevalence ratios were selected as the primary effect measure because several outcomes were common, under which conditions odds ratios may substantially overestimate relative associations. *p*-values for the crude and adjusted prevalence ratios were obtained using two-sided Wald tests based on the estimated regression coefficients and their robust standard errors. These tests evaluated the null hypothesis that the corresponding regression coefficient was equal to zero, equivalent to a prevalence ratio of 1.

Because very small numbers of events were observed in the low-technostress reference category for some outcomes, Firth penalized logistic regression was additionally performed as a sensitivity analysis to address potential sparse-data bias and quasi-complete separation. These penalized models used the same covariate adjustment set as the primary analyses. Firth penalized logistic regression was used exclusively as a sensitivity analysis and was not the source of the *p*-values reported for the primary modified Poisson regression analyses.

Given the unusually large effect estimates observed for some secondary outcomes, additional diagnostic attention was directed to potential methodological explanations for their magnitude. In particular, the distribution of outcome events across technostress categories was examined for sparse-data conditions and quasi-complete separation, while multicollinearity, influential observations, and model convergence were systematically assessed as described below. These diagnostic procedures were intended to evaluate the statistical stability of the models rather than to validate the absolute magnitude of the observed associations.

To assess the robustness of the findings, sensitivity analyses were performed using technostress as a dichotomous variable, comparing high-to-very high technostress with low-to-moderate technostress. Additional analyses evaluated whether the magnitude and direction of the associations remained consistent across the four insulin resistance indicators. Firth penalized logistic regression was applied to the main categorical models as a sensitivity analysis to address sparse-data conditions and potential separation-related issues and to obtain bias-reduced odds ratio estimates [46].

Multicollinearity among candidate covariates was assessed using correlation analyses, variance inflation factors (VIFs), and condition diagnostics. Because occupational social class showed substantial collinearity with educational level, with VIF values exceeding 10 for some social-class categories, social class was excluded from the final multivariable models. The final adjustment set therefore included sex, age as a continuous variable, educational level, physical activity, Mediterranean diet adherence, and smoking status. Potentially influential observations were examined using leverage values and Cook’s distance, and model convergence was verified for all modified Poisson regression models. Correlation coefficients and VIFs are reported in Supplementary Table S2. Only participants with complete information for all study variables were included in the analyses. Therefore, no imputation procedures were performed, and all statistical analyses were conducted using a complete-case approach. Complete-case analysis was used. Of the 105,472 workers initially considered for inclusion, 1297 (1.23%) were excluded because of incomplete information or duplicated/inconsistent records, leaving 104,175 participants (98.77%) in the final analytical sample. Because the proportion excluded was small, multiple imputation was not performed. Individual-level data for excluded workers were not retained in the final analytical dataset; therefore, a comparison of included and excluded participants on baseline characteristics could not be performed.

All hypothesis tests were two-sided, and statistical significance was established at *p* < 0.05. All statistical analyses were performed using IBM SPSS Statistics version 30.0 (IBM Corp., Armonk, NY, USA).

### 2.9. Ethical Considerations

The study was conducted in accordance with the ethical principles of the Declaration of Helsinki and complied with current European and Spanish regulations governing biomedical research and personal data protection [47]. All participants provided written informed consent before inclusion in the occupational health assessment. Data were anonymized before analysis, and only aggregated results are presented.

The study protocol was reviewed and approved by the Research Ethics Committee of the Balearic Islands (Comité de Ética de la Investigación de las Islas Baleares, CEI-IB; reference IB 4383/20; approval date: 26 November 2020).

## 3. Results

### 3.1. Study Population and Distribution of Technostress

Of the 105,472 workers initially considered for inclusion, 1297 (1.23%) were excluded, resulting in a final complete-case analytical sample of 104,175 workers (98.77% of the initially considered population), as shown in Figure 1. Moderate technostress was the most frequent category, observed in 48,513 workers (46.6%), followed by low technostress in 24,258 workers (23.3%), high technostress in 22,901 workers (22.0%), and very high technostress in 8503 workers (8.2%). Overall, 31,404 participants (30.1%) were classified as having high-to-very high technostress.

### 3.2. Sociodemographic and Lifestyle Characteristics According to Technostress Level

Clear sociodemographic and lifestyle differences were observed across technostress categories. The proportion of men increased markedly from 14.5% in the low technostress group to 83.7% in the very high technostress group. Age also showed a strong gradient, with older workers being progressively more represented among those reporting higher levels of technostress.

The detailed distribution of sociodemographic and lifestyle characteristics across technostress categories is presented in Table 1.

Given the magnitude of the differences observed across technostress categories, the coding and distributions of technostress and the principal sociodemographic and lifestyle covariates were re-examined. The values reported in Table 1 and Table 2 were confirmed to reproduce the distributions contained in the analytical dataset, indicating that the marked gradients were not attributable to transcription or tabulation errors. Nevertheless, these pronounced differences demonstrate substantial clustering of technostress with demographic, anthropometric, and lifestyle characteristics and should therefore be considered when interpreting the associations with metabolic outcomes. Standardized mean differences comparing the low and very-high technostress groups confirmed substantial baseline imbalance in several key covariates. The largest differences were observed for physical inactivity (SMD = 2.723), low-to-moderate Mediterranean diet adherence (SMD = 2.552), age (SMD = 2.266), and male sex (SMD = 1.919), whereas the imbalance in current smoking was considerably smaller (|SMD| = 0.126). These findings reinforce the need for cautious interpretation of crude between-group comparisons and careful control of confounding in the multivariable analyses (Supplementary Table S2).

### 3.3. Anthropometric, Biochemical, and Insulin Resistance Markers According to Technostress Level

Anthropometric, biochemical, and insulin resistance markers according to technostress level are presented in Table 2.

A clear graded cross-sectional pattern was observed across technostress categories. Workers reporting higher levels of technostress exhibited progressively less favorable anthropometric and metabolic profiles. Increasing technostress was associated with higher adiposity indicators, elevated fasting glucose and triglyceride concentrations, and lower HDL cholesterol levels.

Similarly, all insulin resistance markers showed a consistent gradient across technostress categories. TyG, TyG-BMI, and METS-IR values increased progressively with rising technostress levels, whereas SPISE values decreased, indicating lower insulin sensitivity among workers experiencing greater technology-related stress.

Overall, higher technostress was associated with progressively less favorable values of the four surrogate insulin resistance indices (Table 2). However, because TyG-BMI, METS-IR, and SPISE incorporate BMI in their calculation, the pronounced differences observed for these indices should be interpreted in the context of the substantial BMI gradient across technostress categories. TyG, which does not incorporate BMI, was therefore considered the primary metabolic outcome.

Importantly, the continuous distributions of the insulin resistance indices showed the same graded cross-sectional pattern across technostress categories. These continuous analyses are particularly informative for TyG-BMI, METS-IR, and SPISE because they are not affected by the extremely small number of participants exceeding the corresponding risk thresholds in the low-technostress reference group and therefore provide a more stable description of the metabolic differences across technostress categories.

### 3.4. Prevalence of Increased Insulin Resistance Risk

The prevalence of increased insulin resistance risk according to technostress level is presented in Table 3.

A marked graded cross-sectional pattern was observed for all insulin resistance markers. The prevalence of increased risk rose progressively across technostress categories, with the highest values consistently observed among workers reporting very high technostress. The strongest gradients were identified for TyG-BMI, METS-IR, and SPISE-IR, whereas TyG also showed a significant but less pronounced increase. All comparisons across technostress categories were statistically significant (*p* < 0.001) (Table 3).

### 3.5. Association Between Technostress and Increased Insulin Resistance Risk

Modified Poisson regression models with robust variance estimation were used to examine the association between technostress level and increased insulin resistance risk. Crude and adjusted prevalence ratios (PRs) with 95% confidence intervals (95% CIs) are presented in Table 4. Adjusted models included sex, age as a continuous variable, educational level, physical activity, Mediterranean diet adherence, and smoking status.

Higher technostress levels were consistently associated with a higher prevalence of increased insulin resistance risk across all markers. A clear graded cross-sectional pattern was observed, with the magnitude of the associations increasing across technostress categories from moderate to very high. These associations remained statistically significant after adjustment for sex, age as a continuous variable, educational level, physical activity, Mediterranean diet adherence, and smoking status.

In the primary analysis, higher technostress was associated with a progressively higher prevalence of increased TyG risk, with adjusted PRs ranging from 1.12 for moderate technostress to 1.77 for very high technostress. Associations were substantially larger for the secondary TyG-BMI, METS-IR, and SPISE-IR outcomes, with some estimates reaching several hundred- or thousand-fold relative to the low-technostress reference category. These exceptionally large estimates should not be interpreted at face value as reliable measures of the magnitude of the association. Their magnitude largely reflects severe sparse-data conditions in the low-technostress reference group, in which only 38 of 24,258 participants met the TyG-BMI risk threshold, 2 of 24,258 met the METS-IR risk threshold, and 6 of 24,258 met the elevated SPISE-IR threshold, resulting in exceptionally large and statistically unstable relative estimates with wide confidence intervals. This extremely low baseline prevalence, together with the pronounced clustering of technostress with adiposity and lifestyle characteristics and the incorporation of BMI into these secondary indices, can produce statistically unstable and exceptionally large relative estimates. Because these sparse-data conditions and potential quasi-complete separation can substantially bias conventional maximum-likelihood logistic regression estimates, Firth penalized logistic regression was applied to the main categorical analyses as a bias-reduced sensitivity analysis using the same covariate adjustment set. The corresponding Firth estimates are reported in Supplementary Table S5 and should be considered when interpreting the categorical secondary outcomes, particularly TyG-BMI, METS-IR, and SPISE-IR. Accordingly, the absolute magnitude of the secondary-outcome estimates should be interpreted with substantial caution, with greater emphasis placed on the direction and consistency of the observed cross-sectional associations.

Diagnostic assessment of the candidate covariates identified substantial collinearity between educational level and occupational social class. VIFs for social class II and III were 17.47 and 19.45, respectively, and educational level and occupational social class were strongly correlated (Spearman ρ = −0.764). Occupational social class was therefore excluded from the final multivariable models. After its exclusion, all VIFs in the final model were below 5. Physical inactivity and poor Mediterranean diet adherence were also strongly correlated (Spearman ρ = 0.856), although their VIFs remained below 5 (4.00 and 3.83, respectively), and both were retained because they represent distinct lifestyle constructs and were prespecified potential confounders. No convergence problems or individual influential observations were identified in the final modified Poisson models. The complete correlation matrix and VIF results are provided in Supplementary Table S3.

Correlations among the four continuous surrogate indices showed that the three BMI-containing indices were very strongly correlated with each other (TyG-BMI and METS-IR: Spearman ρ = 0.979; TyG-BMI and SPISE: ρ = −0.991; METS-IR and SPISE: ρ = −0.979). In contrast, correlations between TyG and the other indices were more moderate (ρ = 0.647 with TyG-BMI, ρ = 0.569 with METS-IR, and ρ = −0.671 with SPISE). These findings indicate substantial redundancy among the BMI-containing indices and support the designation of TyG as the primary outcome, with TyG-BMI, METS-IR, and SPISE considered complementary secondary outcomes. The complete correlation matrix is presented in Supplementary Table S4.

**Figure 2:**
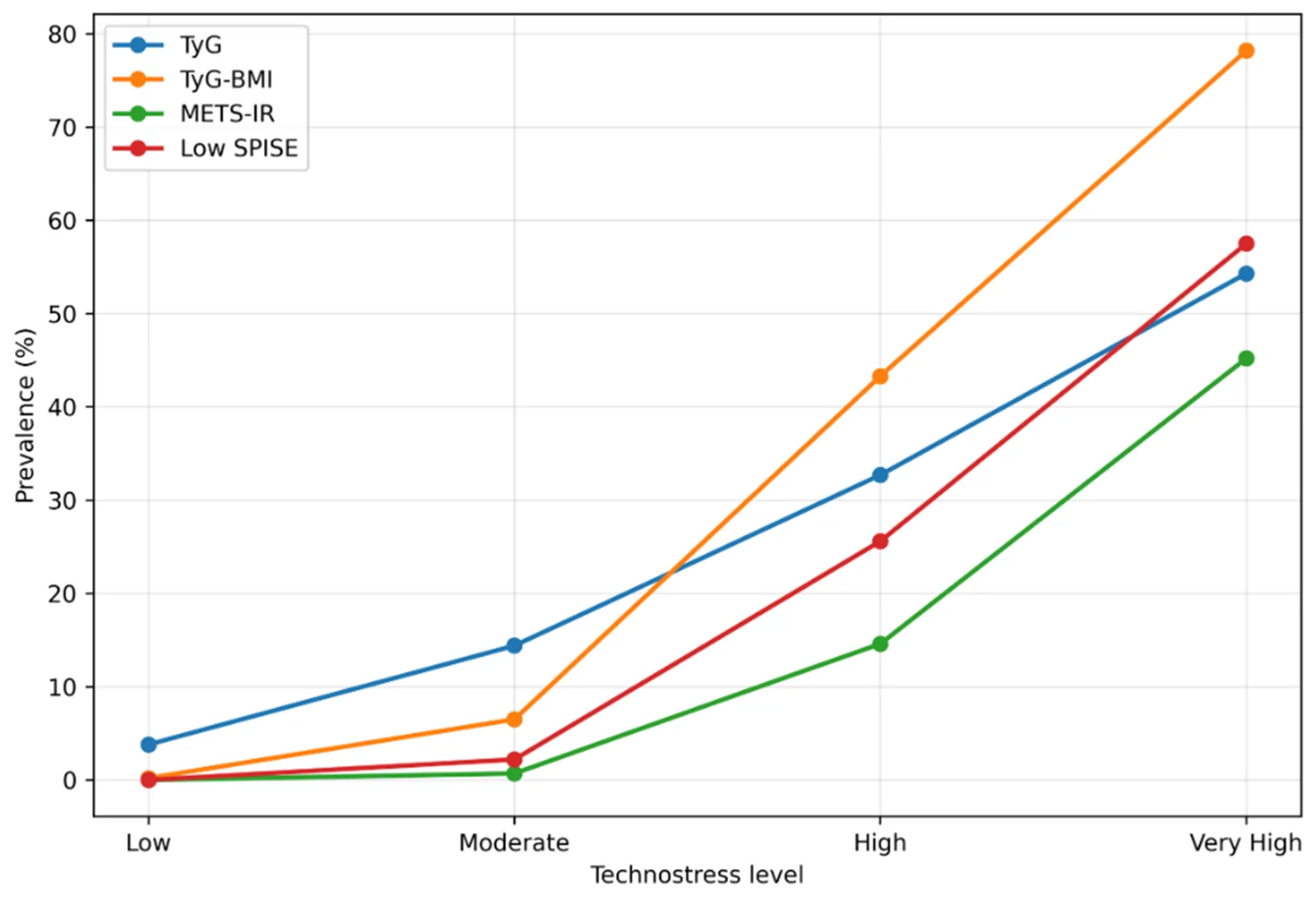
Prevalence of insulin resistance risk according to technostress level.

Figure 2 illustrates the prevalence of insulin resistance risk according to technostress level for the four surrogate markers evaluated in this study.

Prevalence of increased insulin resistance risk across technostress categories according to the four surrogate indices. The curves correspond to increased triglyceride–glucose (TyG) risk, increased triglyceride–glucose body mass index (TyG-BMI) risk, increased metabolic score for insulin resistance (METS-IR) risk, and elevated insulin resistance risk according to the single-point insulin sensitivity estimator (SPISE-IR), as indicated in the figure legend.

### 3.6. Sensitivity Analysis Using Dichotomous Technostress

When technostress was analyzed as a dichotomous variable using modified Poisson regression with robust variance estimation, high-to-very high technostress remained significantly associated with a higher prevalence of all insulin resistance outcomes. Adjusted prevalence ratios are presented in Table 5.

Sensitivity analyses using a dichotomous technostress exposure showed associations in the same direction as those observed in the primary analyses (Table 5). However, the magnitude of the estimates, particularly for the BMI-containing secondary outcomes, remained large and should be interpreted cautiously in view of the pronounced differences in outcome prevalence and participant characteristics between technostress groups. These sensitivity analyses therefore support the consistency of the direction of the observed associations but should not be considered validation of their absolute magnitude.

## 4. Discussion

### 4.1. Principal Findings

The present study examined the relationship between technostress and insulin resistance risk in a large occupational cohort of 104,175 Spanish workers using four validated surrogate markers of insulin resistance: TyG, TyG-BMI, METS-IR, and SPISE. Several relevant findings emerged from the analyses.

First, higher technostress was consistently associated with a higher prevalence of increased insulin resistance risk across all four surrogate indicators. Workers reporting higher levels of technology-related stress exhibited progressively less favorable anthropometric and metabolic profiles, including higher body mass index, greater waist circumference, elevated fasting glucose and triglyceride concentrations, and lower HDL cholesterol levels. These differences were accompanied by less favorable values of the insulin resistance markers. However, because technostress, anthropometric characteristics, and metabolic parameters were assessed cross-sectionally, the temporal direction of these associations cannot be established.

Second, a clear graded cross-sectional pattern was observed for the primary TyG outcome, with the prevalence of increased TyG risk being progressively higher across technostress categories. This pattern should not be interpreted as a dose–response relationship in the causal sense, because exposure and outcome were assessed simultaneously and temporal ordering cannot be established. Similar cross-sectional gradients were observed for the secondary TyG-BMI, METS-IR, and SPISE-IR outcomes. However, these three indices incorporate BMI in their calculation and were very strongly correlated with each other. Their concordant associations therefore should not be regarded as independent confirmation across distinct metabolic domains, as they partly reflect shared information related to adiposity. Accordingly, the TyG findings, which do not incorporate BMI, provide the primary evidence for the observed cross-sectional association, whereas the BMI-containing indices should be interpreted as complementary secondary outcomes.

Third, the associations remained statistically significant after adjustment for multiple sociodemographic and lifestyle factors, including age, sex, educational level, physical activity, adherence to the Mediterranean diet, and smoking status. However, residual confounding remains possible, and the cross-sectional design precludes establishing the temporal direction of the observed associations. Reverse causation is therefore a plausible alternative explanation: workers with obesity, poorer metabolic health, or reduced physical capacity may experience greater difficulty adapting to technological demands and consequently report higher levels of technostress. Thus, the present findings should be interpreted as cross-sectional associations and not as evidence that technostress causes insulin resistance or metabolic deterioration.

The magnitude of several of the observed associations also warrants particular caution. Effect estimates, especially for the BMI-containing secondary indices, were substantially larger than those generally reported in previous epidemiological studies of psychosocial or occupational stress and metabolic outcomes. Such unusually large estimates should not be interpreted as evidence of an exceptionally strong causal effect of technostress. Several characteristics of the present data may have contributed to their magnitude, including the very low prevalence of some outcomes in the low-technostress reference group, the pronounced clustering of technostress with adiposity and lifestyle characteristics, residual confounding, and the incorporation of BMI into TyG-BMI, METS-IR, and SPISE-IR. Sparse-data conditions and potential separation were additionally evaluated using Firth penalized regression. Nevertheless, these analyses cannot eliminate the limitations inherent in the observed data structure. Accordingly, the exceptionally large estimates for the secondary outcomes should be regarded as exploratory and interpreted primarily in terms of the direction and consistency of the cross-sectional associations rather than their absolute magnitude. For these secondary indices, the analyses based on their continuous values provide a more stable and informative description of the observed cross-sectional pattern because they avoid the sparse-data problem created by dichotomization at thresholds with extremely few events in the low-technostress reference group. Accordingly, greater interpretative weight should be placed on the continuous distributions of TyG-BMI, METS-IR, and SPISE than on the absolute magnitude of their categorical relative estimates.

Another noteworthy finding is the pronounced imbalance in demographic, anthropometric, and lifestyle characteristics across technostress categories. Workers with higher technostress were substantially older, more frequently male, had higher BMI, were markedly less physically active, and showed substantially poorer adherence to the Mediterranean diet than workers with low technostress. The magnitude of these differences indicates limited covariate overlap, particularly between the extreme technostress categories. Consequently, although multivariable adjustment was performed, statistical adjustment cannot fully compensate for this limited comparability between exposure groups, and substantial residual confounding may remain. The adjusted estimates should therefore not be interpreted as representing an independent or causal effect of technostress on insulin resistance. Similar clustering of psychosocial stress and unhealthy behaviors has been described in previous occupational health investigations, suggesting the existence of mutually reinforcing pathways linking workplace stressors and metabolic risk [48,49].

### 4.2. Technostress and Insulin Resistance: Potential Biological Mechanisms

Although the present study was not designed to establish causal relationships, several biological and behavioral mechanisms may potentially contribute to the observed association between technostress and insulin resistance risk. The consistency of the findings across four distinct surrogate markers suggests that the observed association may reflect complex interactions involving neuroendocrine, inflammatory, metabolic, and lifestyle-related pathways. However, because of the cross-sectional design, these mechanisms should be regarded as biologically plausible hypotheses rather than causal explanations for the observed associations.

One of the most plausible mechanisms involves chronic activation of the hypothalamic–pituitary–adrenal (HPA) axis. These alterations are recognized precursors of insulin resistance and are central components of the pathophysiological processes leading to type 2 diabetes and metabolic syndrome [50,51].

Beyond neuroendocrine activation, chronic stress has also been linked to a state of persistent low-grade systemic inflammation. Experimental and epidemiological studies have shown that prolonged psychosocial stress is associated with increased circulating concentrations of inflammatory mediators such as interleukin-6, tumor necrosis factor-α, and C-reactive protein [52,53]. Chronic inflammation is therefore considered a key biological bridge connecting psychosocial stressors with metabolic dysfunction [54].

Another potentially relevant pathway involves autonomic nervous system dysregulation. Sustained sympathetic activation contributes to elevated blood pressure, endothelial dysfunction, altered glucose metabolism, and increased insulin resistance [55]. Several studies have reported associations between occupational stress and markers of autonomic imbalance [56].

Sleep disturbances may represent an additional mechanism linking technostress with metabolic impairment. Consequently, individuals experiencing high levels of technostress often report poorer sleep quality and shorter sleep duration [57]. Sleep restriction has consistently been associated with reduced insulin sensitivity, impaired glucose tolerance, increased appetite, hormonal dysregulation, and weight gain [58]. Experimental studies have demonstrated that even short-term sleep deprivation can induce measurable reductions in insulin sensitivity in otherwise healthy individuals [59].

Behavioral mechanisms are likely to play an equally important role. The present study identified substantially lower levels of physical activity and Mediterranean diet adherence among participants with higher technostress levels. Previous investigations have shown that psychosocial stress frequently promotes greater consumption of energy-dense foods rich in refined carbohydrates and saturated fats while simultaneously reducing engagement in health-promoting behaviors [60,61]. Such lifestyle patterns contribute directly to adiposity, dyslipidemia, and insulin resistance.

The observed relationship may also be influenced by the phenomenon of continuous connectivity, often referred to as the ‘always-on’ culture. Modern digital work environments frequently blur the traditional boundaries between professional and personal life, extending work-related cognitive demands beyond conventional working hours [62]. Insufficient recovery has been associated with increased allostatic load, a concept describing the cumulative physiological burden imposed by chronic stress exposure [63].

Taken together, these mechanisms provide biologically plausible pathways that could underlie the observed association between technostress and metabolic health. Although these mechanisms are biologically plausible, the present cross-sectional findings cannot establish that technostress precedes or contributes to the development of insulin resistance. The observed association may also reflect residual confounding, reverse causation, or a bidirectional relationship, and prospective technostress-specific studies are required to clarify temporal ordering.

### 4.3. Sociodemographic and Lifestyle Factors Associated with Technostress and Insulin Resistance

An additional finding of the present study was the pronounced clustering of technostress with several sociodemographic and lifestyle characteristics. Workers reporting higher levels of technology-related stress were older, more frequently male, had lower educational attainment, engaged less often in regular physical activity, and showed substantially poorer adherence to the Mediterranean dietary pattern. Particularly large gradients were observed for physical inactivity and poor Mediterranean diet adherence. These marked differences indicate that technostress was strongly associated with the broader demographic and lifestyle profile of the study population. Accordingly, these characteristics should not be interpreted solely as potential consequences of technostress; they may also represent confounding or shared underlying factors associated with both technostress and metabolic risk.

The association between educational attainment and technostress deserves particular attention. Workers with university-level education were proportionally less represented among those experiencing high and very high technostress. Previous research has suggested that educational level may influence digital literacy, perceived technological self-efficacy, and the ability to adapt to rapidly changing technological environments [64,65].

Occupational social class may also play a relevant role. In the present study, workers belonging to lower occupational categories were disproportionately represented among individuals with higher technostress levels. This observation is consistent with evidence indicating that lower socioeconomic position is frequently associated with reduced job autonomy, lower decision-making capacity, increased job insecurity, and greater exposure to workplace stressors [66].

Lifestyle behaviors showed particularly pronounced differences across technostress categories. Physical inactivity increased markedly with increasing technostress, although the cross-sectional design does not allow the direction of this relationship to be established. Therefore, the observed gradient should not be interpreted as evidence that technostress itself causes reduced physical activity. Given the established relationship between physical inactivity and insulin resistance, careful adjustment for this factor was particularly important in the multivariable analyses [67].

A similarly pronounced pattern was observed for adherence to the Mediterranean diet, with substantially poorer adherence at higher technostress levels. As with physical activity, the cross-sectional design prevents determination of the direction of this association. Consequently, poor Mediterranean diet adherence was treated as a potential confounder in the multivariable analyses, and the observed differences should not be interpreted as evidence that technostress directly causes unhealthy dietary behavior.

Interestingly, the relationship between technostress and smoking was less pronounced than that observed for physical activity or dietary adherence. This finding suggests that the strength of the cross-sectional association with technostress may differ across health behaviors. Nevertheless, tobacco use remains an important contributor to insulin resistance and cardiovascular disease and may interact with other lifestyle factors in determining overall metabolic risk [68].

Taken together, these observations support the concept that technostress should be considered within a broader biopsychosocial framework.

### 4.4. Comparison with Previous Studies

Direct comparisons between the present findings and previous investigations are challenging because research specifically examining the relationship between technostress and insulin resistance remains extremely limited. Most studies conducted to date have focused primarily on the psychological, organizational, and behavioral consequences of technology-related stress, whereas its potential metabolic implications have received considerably less attention. Consequently, the current study contributes novel evidence to an emerging field of occupational health research by demonstrating consistent associations between technostress and multiple validated markers of insulin resistance in a very large working population.

Previous investigations have consistently linked technostress with adverse psychological outcomes, including burnout, emotional exhaustion, anxiety, depressive symptoms, reduced work engagement, and diminished job satisfaction [69,70,71]. Chronic activation of stress-response systems, emotional strain, and impaired recovery have been proposed as central mechanisms through which technology-related stress affects overall health [72]. The present findings extend this perspective by identifying a cross-sectional association between technostress and metabolic dysfunction; however, the temporal direction and causal nature of this relationship cannot be established from the present data.

The present findings should be interpreted in the context of the prospective literature on occupational stress and cardiometabolic health. Previous longitudinal studies have reported associations between chronic psychosocial work stress and subsequent metabolic syndrome, type 2 diabetes, central obesity, and cardiovascular disease [73,74,75,76]. However, the effect sizes reported in previous prospective studies of psychosocial and occupational stress have generally been considerably more modest than those observed in the present study. For several of our secondary outcomes, the estimated associations exceed those typically reported in the previous literature by several orders of magnitude. This exceptional discrepancy requires particular caution and argues against interpreting the absolute magnitude of these estimates as reflecting the causal effect of technostress on metabolic risk. This difference is important when interpreting our findings and may reflect, at least in part, the pronounced clustering of technostress with obesity, lifestyle characteristics, and other sociodemographic factors in this population, as well as residual confounding and possible reverse causation. Therefore, the magnitude of the associations observed here should not be interpreted as evidence that technostress exerts a correspondingly large causal effect on insulin resistance. Rather, the prospective occupational stress literature provides broader contextual support for a potential relationship between work-related psychosocial exposures and metabolic health, while longitudinal technostress-specific studies with more comprehensive assessment of occupational and organizational characteristics are needed to establish temporality and better characterize the observed association.

Another relevant comparison can be made with studies investigating burnout and metabolic health. Burnout is increasingly recognized as a condition associated not only with psychological impairment but also with systemic physiological alterations. Recent evidence suggests that individuals experiencing burnout exhibit higher levels of inflammatory biomarkers, greater cardiometabolic risk, and less favorable metabolic profiles [77,78].

The results are also broadly consistent with recent occupational studies examining insulin resistance in different working populations. Several investigations conducted in Spanish workers have reported significant associations between insulin resistance markers and occupational characteristics, lifestyle habits, social determinants, and work organization factors [79,80,81]. Although previous studies from our research group have evaluated TyG, TyG-BMI, METS-IR, and SPISE in large samples of Spanish workers in relation to occupational and lifestyle factors, the specific contribution of the present analysis is the evaluation of these established metabolic indices in relation to technostress as the exposure of interest.

Particularly noteworthy is the consistency observed across all four insulin resistance indicators. Although the magnitude of the associations differed according to the marker employed, the direction of the relationship remained consistent. Nevertheless, given the cross-sectional design and the potential for residual confounding, these findings should not be interpreted as evidence of a causal biological effect of technostress on metabolic regulation.

Overall, the present study expands the existing literature by providing evidence that technostress may represent a previously underrecognized factor associated with insulin resistance risk. Although longitudinal studies are required to clarify temporal relationships and causal pathways, the findings align with current knowledge regarding occupational stress and metabolic health while highlighting a cross-sectional association between technostress and cardiometabolic risk in increasingly digitalized workplaces.

### 4.5. Clinical and Occupational Health Implications

The findings of the present study may have important implications for both clinical practice and occupational health management. Traditionally, technostress has been viewed primarily as a psychosocial phenomenon affecting employee well-being, job satisfaction, productivity, and mental health. However, the results reported here indicate that higher technostress is cross-sectionally associated with less favorable metabolic profiles, suggesting that its relationship with metabolic health warrants further investigation. Given the increasing prevalence of digital technologies in contemporary workplaces, this observation deserves careful consideration from healthcare professionals, employers, and policy makers.

From a clinical perspective, the consistent associations observed between technostress and multiple insulin resistance markers suggest that psychosocial and digital work-related factors may warrant consideration in the broader assessment of cardiometabolic risk. Current prevention strategies for insulin resistance and type 2 diabetes largely focus on traditional risk factors such as obesity, physical inactivity, unhealthy dietary habits, smoking, and family history [82]. While these determinants remain fundamental, the present findings indicate that higher technostress is cross-sectionally associated with less favorable metabolic profiles. Given the inability to establish temporality or causality, technostress should be considered a potential occupational correlate of metabolic risk rather than a demonstrated contributor to metabolic dysfunction.

The results also have important implications for occupational health services. Organizational interventions aimed at reducing technostress may include improving digital literacy, providing adequate training before implementing new technologies, enhancing technical support, establishing clear communication protocols, and promoting realistic expectations regarding digital availability and response times [83]. Such measures may improve psychological well-being, whereas their potential effects on metabolic health outcomes require evaluation in prospective and intervention studies.

Particular attention should be paid to the phenomenon of constant connectivity. Several authors have suggested that the inability to psychologically disconnect from work may impair recovery processes and increase long-term health risks [84].

The identification of particularly vulnerable groups is another relevant implication of the present study. Workers with lower educational attainment, lower occupational social class, reduced physical activity, and poorer dietary habits appeared more likely to experience elevated levels of technostress.

From a broader public health perspective, the findings contribute to an expanding body of evidence showing associations between occupational environments and metabolic health.

### 4.6. Strengths and Limitations

Several strengths of this study should be acknowledged. First, the investigation was conducted in a very large occupational cohort comprising more than 104,000 workers from different economic sectors and occupational categories. The large sample size provided substantial statistical power and enabled the detection of consistent associations across multiple analyses. In addition, the inclusion of workers with diverse sociodemographic characteristics increases the external validity of the findings and enhances their relevance to contemporary working populations.

A second strength is the evaluation of several validated surrogate markers of insulin resistance. However, these markers should not be considered statistically or biologically independent. TyG was designated as the primary outcome because it does not incorporate BMI, whereas TyG-BMI, METS-IR, and SPISE-IR were considered complementary secondary outcomes because BMI contributes directly to their calculation. The very strong correlations observed among these three indices confirm substantial shared information related to adiposity. Given the pronounced BMI gradient across technostress categories, part of the stronger associations observed for TyG-BMI, METS-IR, and SPISE-IR may therefore reflect differences in adiposity rather than independent metabolic information. Accordingly, the association observed for TyG provides the principal evidence regarding insulin resistance risk, while findings for the BMI-containing indices should be interpreted as supportive but not independent evidence.

Third, the analyses incorporated a wide range of potential confounding variables, including age, sex, educational level, smoking status, physical activity, and adherence to the Mediterranean diet. Occupational social class was initially considered as a candidate covariate but was excluded from the final multivariable models because of substantial collinearity with educational level. Although adjustment for the retained covariates was performed, the pronounced covariate imbalance and limited overlap across technostress categories restrict the ability of multivariable regression to disentangle the association of technostress from the accompanying demographic, anthropometric, and lifestyle differences. Therefore, substantial residual confounding may remain, and the adjusted estimates should not be interpreted as representing an independent effect of technostress. Moreover, sensitivity analyses using alternative specifications of technostress and Firth penalized regression yielded associations in the same general direction as the primary analyses. However, these analyses should not be interpreted as validating the absolute magnitude of the exceptionally large estimates observed for some secondary outcomes, which remain susceptible to the influence of sparse reference-group events, pronounced clustering with adiposity and lifestyle characteristics, and residual confounding.

The study also addresses a topic of increasing relevance in occupational medicine. While the psychological consequences of technostress have been extensively investigated, considerably less attention has been devoted to its potential metabolic implications. To our knowledge, this is one of the largest studies to explore the relationship between technostress and insulin resistance using multiple validated surrogate markers. Consequently, the findings contribute novel evidence to an area that remains relatively underexplored despite the growing digitalization of modern workplaces.

Despite these strengths, several limitations should be considered when interpreting the results. The most important limitation is the cross-sectional design. Because exposure and outcome were assessed simultaneously, temporal relationships cannot be established and causal inferences cannot be drawn. Although the observed associations are biologically plausible and remained significant after extensive adjustment, it is not possible to determine whether technostress contributed to the development of insulin resistance or whether individuals with poorer metabolic health were more likely to perceive greater technology-related stress. Longitudinal studies will be necessary to clarify the directionality of these relationships. In addition, individual-level data for excluded workers were not retained in the final analytical dataset, precluding a direct comparison of baseline characteristics between included and excluded participants. However, exclusions represented only 1.23% of the initially considered population, with 98.77% retained in the final analytical sample.

A further statistical limitation concerns the small number of high-risk events in the low-technostress reference category for some insulin resistance indicators. This sparse-data structure may lead to unstable relative estimates, particularly for TyG-BMI, METS-IR, and SPISE-IR. To reduce the impact of this issue, prevalence ratios estimated using modified Poisson regression with robust variance were used as the primary effect measures, and Firth penalized logistic regression was performed as a sensitivity analysis to address sparse-data bias and potential quasi-complete separation. Diagnostic assessment of the revised models did not indicate convergence problems or individual influential observations. Substantial multicollinearity identified between educational level and occupational social class was addressed by excluding occupational social class from the final multivariable models, after which all VIF values were below 5. Nevertheless, the magnitude of the associations for outcomes with very few reference-group events should be interpreted cautiously.

A further limitation concerns the unusually large magnitude of some effect estimates, particularly for the BMI-containing secondary outcomes. These estimates exceed those generally reported in previous studies of psychosocial or occupational stress and metabolic outcomes by several orders of magnitude and should therefore not be interpreted at face value as measures of causal effect. The very low number of events in the low-technostress reference group for some outcomes, together with the pronounced clustering of technostress with adiposity and lifestyle characteristics and the BMI dependence of the secondary indices, likely contributed to these extreme relative estimates. Although penalized regression and model diagnostics were used to evaluate sparse-data bias, separation, multicollinearity, influential observations, and convergence, these procedures cannot establish that the absolute magnitude of the observed associations reflects the underlying etiological relationship.

Another limitation concerns the assessment of technostress. The 15-item short-form questionnaire used in this study covered the five commonly described dimensions of technostress; however, this specific short form has not undergone complete external psychometric validation. Moreover, the analytical dataset retained only the final technostress category and not the underlying individual continuous score or item-level responses. Consequently, technostress could not be analyzed as a continuous exposure, and internal consistency and factorial structure could not be retrospectively evaluated. Test–retest reliability and concurrent validity against an established technostress instrument were also unavailable. In addition, the four technostress categories used in the present study should be regarded as predefined analytical groupings rather than externally validated diagnostic thresholds. Therefore, we could not determine whether the observed associations were independent of these pragmatic category boundaries, and the graded pattern observed across categories should not be interpreted as evidence of a linear dose–response relationship. These measurement-related limitations should be considered when interpreting the magnitude of the observed associations and highlight the need for future studies using fully validated technostress instruments.

The use of surrogate markers rather than direct measurements of insulin sensitivity represents an additional limitation. Techniques such as the hyperinsulinemic–euglycemic clamp remain the gold standard for assessing insulin resistance but are impractical in large epidemiological studies. However, the indices employed in the present investigation have been extensively validated and have demonstrated good performance for identifying individuals at increased metabolic risk. Nevertheless, the concordance of findings across the four markers should not be interpreted as independent confirmation, because TyG-BMI, METS-IR, and SPISE are very strongly correlated and share BMI and other metabolic components. Accordingly, TyG provides the primary evidence regarding insulin-resistance risk, whereas the findings for TyG-BMI, METS-IR, and SPISE should be considered supportive but not independent evidence.

Residual confounding cannot be completely excluded. Although several important sociodemographic and lifestyle factors were included in the analyses, occupational social class was excluded from the final multivariable models because of substantial collinearity with educational level. In addition, the pronounced gradients observed across technostress categories for sex, BMI, physical activity, and Mediterranean diet adherence indicate substantial clustering of technostress with demographic, anthropometric, and lifestyle characteristics. Although adjustment attenuated the associations and the final models did not show problematic VIF values, residual confounding and the possibility that technostress partly captures a broader underlying sociodemographic or lifestyle profile cannot be excluded. Information regarding other potentially relevant occupational and individual-level variables, including working hours, shift work, occupational sector, actual intensity of ICT use, teleworking, job demands, job control, job strain, employment contract characteristics, organizational factors, sleep quality, income level, psychological disorders, medication use, and family history of metabolic disease, was not available in the analytical dataset. Because several of these occupational characteristics could be associated with both perceived technostress and metabolic health, unmeasured occupational confounding cannot be excluded. The exceptionally large baseline differences in age, sex, physical activity, and Mediterranean diet adherence across technostress categories further increase the potential for residual confounding, even after multivariable adjustment. Although age was modeled as a continuous variable in the revised analyses, physical activity and Mediterranean diet adherence could only be included as dichotomous covariates because the original continuous IPAQ and MEDAS scores were not retained in the analytical dataset. Therefore, incomplete control of lifestyle-related confounding remains possible, and the adjusted estimates should not be interpreted as demonstrating an effect of technostress independent of these characteristics. Consequently, the observed associations should be interpreted cautiously and should be confirmed in prospective studies with more comprehensive assessment of potential confounding factors.

Finally, the study population consisted exclusively of Spanish workers. Cultural, occupational, and organizational characteristics may differ across countries and labor markets, potentially limiting the generalizability of the findings to other settings. Replication in different populations and occupational environments will therefore be important to determine the broader applicability of the results.

Overall, the large sample size and comprehensive metabolic assessment are important strengths of the study. Nevertheless, the cross-sectional design, the pronounced clustering of technostress with adiposity and lifestyle characteristics, the possibility of residual confounding, and potential reverse causation require cautious interpretation of the observed associations. Prospective studies with comprehensive assessment of occupational, organizational, sociodemographic, and lifestyle factors are needed to establish temporal ordering and to determine whether technostress is prospectively associated with subsequent changes in metabolic health.

### 4.7. Future Research Directions

The findings of the present study open several avenues for future research. First, prospective longitudinal studies are needed to establish the temporal relationship between technostress and insulin resistance or other cardiometabolic abnormalities. The cross-sectional design of the present study does not allow determination of whether technostress precedes metabolic alterations or whether individuals with obesity, poorer metabolic health, or reduced physical function are more likely to perceive greater difficulty in coping with workplace technology. Longitudinal analyses with repeated assessment of both technostress and metabolic outcomes are therefore needed to distinguish between these possibilities and to determine whether the observed relationship is prospective, reverse, or bidirectional.

Future studies should incorporate a more comprehensive assessment of occupational and organizational characteristics that were unavailable in the present study, including working hours, shift work, occupational sector, intensity and frequency of ICT use, teleworking, job demands, job control, job strain, employment contract characteristics, and organizational factors. These variables may be associated with both perceived technostress and metabolic health and should therefore be considered as potential confounders in longitudinal analyses.

Another important area for future research concerns the impact of emerging digital work environments. The rapid expansion of remote work, hybrid work models, artificial intelligence systems, digital monitoring technologies, and algorithm-driven management practices is transforming occupational settings worldwide [85].

Intervention studies should also be prioritized. Randomized workplace interventions aimed at improving digital competencies, enhancing organizational support, promoting healthy technology use, and facilitating psychological detachment from work could help determine whether reducing technostress leads to measurable improvements in metabolic health outcomes [86].

Future investigations may additionally benefit from examining potential differences according to sex, age, occupational sector, and socioeconomic position. Associations between technostress and health outcomes may differ across worker subgroups, and certain groups may show greater vulnerability to adverse health profiles associated with higher technostress.

Finally, research integrating mental health, occupational exposures, and metabolic outcomes may provide a more comprehensive understanding of worker health in increasingly digitalized societies. Traditional approaches have often considered psychological and metabolic disorders separately; however, growing evidence indicates that these conditions are closely interconnected and may share common determinants and biological pathways [87].

## 5. Conclusions

The present study found that higher technostress was associated with a higher prevalence of increased insulin resistance risk according to TyG, the primary outcome, in a large cohort of Spanish workers. A graded cross-sectional pattern was observed across technostress categories, and the association remained significant after adjustment for major sociodemographic and lifestyle factors. Associations were also observed for the secondary BMI-containing indices (TyG-BMI, METS-IR, and SPISE-IR), although their substantially larger estimates and strong intercorrelations warrant cautious interpretation.

Given the cross-sectional design, these findings do not establish temporality or causality and may be influenced by residual confounding or reverse causation. Prospective studies with repeated assessments of technostress and metabolic outcomes are needed to determine the temporal direction and clinical relevance of these associations.

## Figures and Tables

**Table 1:** Characteristics of the study population according to technostress level.

Variable	Low (*n* = 24,258)	Moderate (*n* = 48,513)	High (*n* = 22,901)	Very High (*n* = 8503)	*p*-Value
Sex					<0.001
Men	3511 (14.5)	34,148 (70.4)	18,100 (79.0)	7117 (83.7)	
Women	20,747 (85.5)	14,365 (29.6)	4801 (21.0)	1386 (16.3)	
Age group (years)					<0.001
20–29	7080 (29.2)	10,245 (21.1)	1846 (8.1)	170 (2.0)	
30–39	11,386 (46.9)	17,586 (36.3)	4992 (21.8)	605 (7.1)	
40–49	5426 (22.4)	15,062 (31.0)	7804 (34.1)	2502 (29.4)	
50–59	365 (1.5)	5244 (10.8)	6840 (29.9)	4048 (47.6)	
60–69	1 (0.0)	376 (0.8)	1419 (6.2)	1178 (13.9)	
Educational level					<0.001
Primary	7056 (29.1)	30,517 (62.9)	16,032 (70.0)	6139 (72.2)	
Secondary	14,083 (58.1)	15,886 (32.7)	6152 (26.9)	2168 (25.5)	
University	3119 (12.9)	2110 (4.3)	717 (3.1)	196 (2.3)	
Social class					<0.001
Class I	3048 (12.6)	2261 (4.7)	758 (3.3)	213 (2.5)	
Class II	11,818 (48.7)	9047 (18.6)	2973 (13.0)	963 (11.3)	
Class III	9392 (38.7)	37,205 (76.7)	19,170 (83.7)	7327 (86.2)	
Physical activity					<0.001
No	4171 (17.2)	22,321 (46.0)	19,420 (84.8)	8246 (97.0)	
Yes	20,087 (82.8)	26,192 (54.0)	3481 (15.2)	257 (3.0)	
Mediterranean diet adherence					<0.001
No	4849 (20.0)	24,473 (50.4)	19,721 (86.1)	8289 (97.5)	
Yes	19,409 (80.0)	24,040 (49.6)	3180 (13.9)	214 (2.5)	
Current smoking					<0.001
No	16,072 (66.3)	30,071 (62.0)	14,912 (65.1)	6129 (72.1)	
Yes	8186 (33.7)	18,442 (38.0)	7989 (34.9)	2374 (27.9)	

Values are expressed as *n* (%). *p*-values were obtained using Pearson’s chi-square test. Overall, higher technostress levels were associated with older age, lower educational attainment, lower occupational social class, reduced physical activity, poorer adherence to the Mediterranean diet, and a less favorable lifestyle profile. All comparisons across technostress categories were statistically significant (*p* < 0.001).

**Table 2:** Anthropometric, biochemical, and insulin resistance markers according to technostress level.

Variable	Low (*n* = 24,258)	Moderate (*n* = 48,513)	High (*n* = 22,901)	Very High (*n* = 8503)	*p*-Value
Age (years)	33.94 ± 7.03	37.73 ± 9.22	44.99 ± 10.04	51.25 ± 8.20	<0.001
BMI (kg/m^2^)	22.32 ± 2.35	25.31 ± 3.09	29.49 ± 4.14	32.61 ± 4.65	<0.001
Waist circumference (cm)	71.90 ± 6.59	81.51 ± 7.67	89.87 ± 9.91	95.32 ± 11.08	<0.001
Fasting glucose (mg/dL)	81.90 ± 9.97	84.99 ± 10.82	89.24 ± 11.81	100.70 ± 17.07	<0.001
Triglycerides (mg/dL)	77.14 ± 33.58	101.52 ± 61.03	139.24 ± 94.70	170.11 ± 116.57	<0.001
HDL cholesterol (mg/dL)	55.45 ± 7.10	52.57 ± 6.82	49.42 ± 6.96	46.93 ± 7.20	<0.001
TyG index	7.97 ± 0.41	8.20 ± 0.49	8.54 ± 0.56	8.87 ± 0.60	<0.001
TyG-BMI	178.08 ± 22.20	207.87 ± 32.54	252.59 ± 45.28	289.29 ± 44.93	<0.001
METS-IR	30.55 ± 3.72	36.77 ± 5.07	44.56 ± 6.94	50.01 ± 7.77	<0.001
SPISE	8.60 ± 1.49	7.31 ± 1.46	5.55 ± 1.28	4.43 ± 1.02	<0.001

Values are expressed as mean ± standard deviation (SD). *p*-values were obtained using one-way analysis of variance (ANOVA).

**Table 3:** Prevalence of increased insulin resistance risk according to technostress level.

Outcome	Low (*n* = 24,258)	Moderate (*n* = 48,513)	High (*n* = 22,901)	Very High (*n* = 8503)	*p*-Value
Increased TyG risk	921 (3.8)	6967 (14.4)	7499 (32.7)	4616 (54.3)	<0.001
Increased TyG-BMI risk	38 (0.2)	3166 (6.5)	9905 (43.3)	6652 (78.2)	<0.001
Increased METS-IR risk	2 (0.0)	317 (0.7)	3335 (14.6)	3840 (45.2)	<0.001
Elevated SPISE-IR (high IR risk)	6 (0.0)	1073 (2.2)	5863 (25.6)	4890 (57.5)	<0.001

Values are expressed as *n* (%). *p*-values were obtained using Pearson’s chi-square test.

**Table 4:** Crude and adjusted prevalence ratios for increased insulin resistance risk according to technostress level.

Outcome	Technostress Level	Crude PR (95% CI)	*p*-Value	Adjusted PR (95% CI)	*p*-Value
Increased TyG risk	Low	1.00 Reference	—	1.00 Reference	—
	Moderate	3.78 (3.54–4.04)	<0.001	1.12 (1.05–1.20)	<0.001
	High	8.62 (8.07–9.21)	<0.001	1.28 (1.19–1.38)	<0.001
	Very high	14.30 (13.38–15.28)	<0.001	1.77 (1.64–1.91)	<0.001
Increased TyG-BMI risk	Low	1.00 Reference	—	1.00 Reference	—
	Moderate	41.66 (30.27–57.34)	<0.001	19.72 (14.37–27.05)	<0.001
	High	276.10 (200.88–379.49)	<0.001	84.35 (61.61–115.50)	<0.001
	Very high	499.40 (363.40–686.30)	<0.001	171.73 (125.34–235.27)	<0.001
Increased METS-IR risk	Low	1.00 Reference	—	1.00 Reference	—
	Moderate	79.25 (19.74–318.26)	<0.001	50.86 (12.76–202.71)	<0.001
	High	1766.31 (441.61–7064.76)	<0.001	868.38 (219.20–3440.14)	<0.001
	Very high	5477.52 (1369.69–21,905.17)	<0.001	3730.57 (941.20–14,786.58)	<0.001
Elevated SPISE-IR (high IR risk)	Low	1.00 Reference	—	1.00 Reference	—
	Moderate	89.42 (40.09–199.46)	<0.001	47.53 (21.53–104.92)	<0.001
	High	1035.07 (464.91–2304.45)	<0.001	379.64 (172.88–833.67)	<0.001
	Very high	2325.09 (1044.44–5176.02)	<0.001	1052.09 (479.01–2310.78)	<0.001

Adjusted models included sex, age as a continuous variable, educational level, physical activity, Mediterranean diet adherence, and smoking status. Low technostress was used as the reference category. PR: prevalence ratio; CI: confidence interval; IR: insulin resistance. *p*-values for crude and adjusted prevalence ratios were obtained using two-sided Wald tests based on the estimated regression coefficients and their robust standard errors.

**Table 5:** Sensitivity analyses using dichotomous technostress exposure.

Outcome	Low-to-Moderate Technostress *n* (%)	High-to-Very High Technostress *n* (%)	Adjusted PR (95% CI)	*p*-Value
Increased TyG risk	10.8	38.6	1.26 (1.23–1.30)	<0.001
Increased TyG-BMI risk	4.4	52.7	5.65 (5.47–5.85)	<0.001
Increased METS-IR risk	0.4	22.8	29.58 (26.49–33.04)	<0.001
Elevated SPISE-IR (high IR risk)	1.5	34.2	11.73 (11.04–12.46)	<0.001

Adjusted for sex, age as a continuous variable, educational level, physical activity, Mediterranean diet adherence, and smoking status. PR: prevalence ratio; CI: confidence interval; IR: insulin resistance. *p*-values were obtained using two-sided Wald tests based on the estimated regression coefficients and their robust standard errors.

## Data Availability

The original contributions presented in this study are included in the article/Supplementary Material. Further inquiries can be directed to the corresponding author(s).

## Supplementary Materials

The following supporting information can be downloaded at https://www.mdpi.com/article/10.3390/medsci14050545/s1. Supplementary Table S1. Complete 15-item technostress questionnaire used in the study. Supplementary Table S2. Standardized differences in selected participant characteristics between the low and very-high technostress groups. Supplementary Table S3. Correlations among technostress and study covariates and variance inflation factors for the final multivariable model. Supplementary Table S4. Spearman correlations among surrogate indices of insulin resistance. Supplementary Table S5. Sensitivity analysis using Firth penalized logistic regression for the association between technostress level and increased insulin resistance risk.
