# Supplementary material for: Technostress and Insulin Resistance Risk: A Cross-Sectional Analysis of 104,175 Spanish Workers Using TyG, TyG-BMI, METS-IR, and SPISE Indices
Source: Med Sci (Basel). 2026 Sep 3;14(5):545. doi: 10.3390/medsci14050545 (PMC13609671; doi:10.3390/medsci14050545)
Supplement: Supplementary file 1 [file medsci-14-00545-s001.zip › medsci-4495348-supplementary.pdf]

Supplementary Materials

Technostress and Insulin Resistance Risk: A Cross-Sectional Analysis of 104,175 Spanish Workers Using TyG, TyG-BMI, METS-IR, and SPISE Indices

Supplementary Table S1. Complete 15-item technostress questionnaire used in the study.

Dimension	Item	Questionnaire statement
**Techno-overload**	1	Digital technologies require me to work faster than I can manage.
	2	Digital tools increase my workload.
	3	I feel pressured by having to deal with too many digital communications.
**Techno-invasion**	4	I find it difficult to disconnect from work because of technology.
	5	I feel that I have to be digitally available outside working hours.
	6	Technologies intrude into my personal life.
**Techno-complexity**	7	I need to spend a considerable amount of time learning new digital tools.
	8	Some of the technologies I use are too complex.
	9	I sometimes feel that my digital skills are insufficient.
**Techno-uncertainty**	10	Technologies are constantly changing in my workplace.
	11	I have to continuously adapt to new platforms or systems.
	12	Technological changes create uncertainty in my work.
**Techno-insecurity**	13	I am concerned that people with better digital skills could replace me.
	14	I feel pressure to continuously update my technological skills.
	15	Automation or artificial intelligence could negatively affect my job.

Response scale: All items were rated on a 5-point Likert scale: **1 = strongly disagree, 2 = disagree, 3 = neither agree nor disagree, 4 = agree, and 5 = strongly agree.**

Scoring: The occupational assessment system calculated the overall technostress score as the arithmetic mean of the 15 items, yielding a score ranging from 1 to 5, with higher scores indicating greater perceived technostress. The resulting score was classified into the following operational categories: low (1.0–2.0), moderate (2.1–3.0), high (3.1–4.0), and very high (4.1–5.0). The analytical dataset retained only the final technostress category and not the underlying individual continuous score or item-level responses.

Note: These score ranges were used as operational analytical categories and should not be interpreted as externally validated clinical or diagnostic thresholds.

Supplementary Table S2. Standardized differences in selected participant characteristics between the low and very-high technostress groups.

Characteristic	Low technostress (n = 24,258)	Very-high technostress (n = 8,503)	SMD
Age, years	33.94 ± 7.03	51.25 ± 8.20	2.266
Male sex, %	14.5	83.7	1.919
Physical inactivity, %	17.2	97.0	2.723
Low-to-moderate Mediterranean diet adherence, %	20.0	97.5	2.552
Current smoking, %	33.7	27.9	−0.126

Values are expressed as mean ± standard deviation or percentage, as appropriate. SMD, standardized mean difference. SMDs compare the very-high technostress group with the low-technostress group. Positive values indicate higher values or prevalence in the very-high technostress group, whereas negative values indicate lower prevalence. Absolute SMD values ≥0.10 were considered indicative of meaningful imbalance.

Supplementary Table S3. Correlations among technostress and study covariates and variance inflation factors for the final multivariable model.

Panel A. Spearman correlation matrix

Variable	1	2	3	4	5	6	7	8
1. Technostress	1.000							
2. Male sex	0.460	1.000						
3. Age group	0.449	0.020	1.000					
4. Educational level	−0.295	−0.097	−0.044	1.000				
5. Occupational social class	0.339	0.179	0.050	**−0.764**	1.000			
6. Physical inactivity	0.527	0.067	0.283	−0.070	0.083	1.000		
7. Poor Mediterranean diet adherence	0.514	0.104	0.270	−0.067	0.084	**0.856**	1.000	
8. Current smoking	−0.016	0.046	−0.071	−0.113	0.110	−0.038	−0.035	1.000

Panel B. Variance inflation factors in the final multivariable model

Predictor	VIF
Moderate technostress	2.79
High technostress	3.54
Very high technostress	2.57
Male sex	1.57
Age 30–39 years	1.89
Age 40–49 years	1.97
Age 50–59 years	1.96
Age 60–69 years	1.28
Secondary education	1.18
University education	1.12
Physical inactivity	4.00
Poor Mediterranean diet adherence	3.83
Current smoking	1.02

Spearman correlation coefficients are presented in Panel A. Technostress and ordinal covariates were coded in increasing category order; physical inactivity, poor Mediterranean diet adherence, male sex, and current smoking were coded as binary variables. Occupational social class was included in the correlation matrix because it was initially considered as a candidate covariate but was excluded from the final multivariable models because of substantial collinearity with educational level. Before its exclusion, VIFs were 17.47 and 19.45 for social class II and III, respectively. Panel B presents VIFs after exclusion of occupational social class. Reference categories for dummy-coded variables were low technostress, female sex, age 20–29 years, and primary education.

Supplementary Table S4. Spearman correlations among surrogate indices of insulin resistance.

Index	TyG	TyG-BMI	METS-IR	SPISE
**TyG**	1.000	0.647	0.569	−0.671
**TyG-BMI**	0.647	1.000	0.979	−0.991
**METS-IR**	0.569	0.979	1.000	−0.979
**SPISE**	−0.671	−0.991	−0.979	1.000

Values are Spearman correlation coefficients. All correlations were statistically significant ($p < 0.001$). TyG-BMI, METS-IR, and SPISE incorporate BMI in their calculation, whereas TyG does not. The negative correlations involving SPISE reflect that higher SPISE values indicate greater insulin sensitivity, whereas higher values of the other indices indicate greater insulin resistance.

Supplementary Table S5. Sensitivity analysis using Firth penalized logistic regression for the association between technostress level and increased insulin resistance risk.

Outcome	Technostress	Firth adjusted OR (95% CI)
**TyG**	Moderate	1.07 (0.98–1.16)
	High	1.32 (1.20–1.44)
	Very high	2.51 (2.26–2.79)
**TyG-BMI**	Moderate	40.95 (29.65–56.56)
	High	528.26 (381.02–732.41)
	Very high	4461.89 (3188.52–6243.78)
**METS-IR**	Moderate	62.20 (17.95–215.58)
	High	1972.76 (570.62–6820.27)
	Very high	22012.74 (6347.52–76338.52)
**SPISE-IR**	Moderate	69.69 (32.19–150.90)
	High	1138.60 (525.67–2466.21)
	Very high	8254.53 (3797.37–17943.29)

Adjusted models included sex, age as a continuous variable, educational level, physical activity, Mediterranean diet adherence, and smoking status. Low technostress was used as the reference category. Firth penalized logistic regression was used to reduce sparse-data bias and address potential quasi-complete separation arising from the very small number of outcome events in the low-technostress reference category. Despite penalization, estimates for the secondary outcomes remained exceptionally large and should therefore be interpreted cautiously, with greater emphasis on the direction of the associations than on their absolute magnitude. OR: odds ratio; CI: confidence interval; IR: insulin resistance.
